# Effects of Probiotics on Intermediate Cardiovascular Outcomes in Patients with Overweight or Obesity: A Systematic Review and Meta-Analysis

**DOI:** 10.3390/jcm12072554

**Published:** 2023-03-28

**Authors:** Frank Mayta-Tovalino, Carlos Diaz-Arocutipa, Alejandro Piscoya, Adrian V. Hernandez

**Affiliations:** 1 Unidad de Revisiones Sistemáticas y Meta-Análisis (URSIGET), Vicerrectorado de Investigación, Universidad San Ignacio de Loyola, Av. La Fontana 550, Lima 15024, Peru; 2 Health Outcomes, Policy and Evidence Synthesis (HOPES) Group, University of Connecticut School of Pharmacy, 69 N Eagleville Rd U-3092, Storrs, CT 06269, USA

**Keywords:** overweight, obesity, probiotics, meta-analysis

## Abstract

Background: Clinical trials evaluating the effect of probiotics on cardiovascular intermediate outcomes have been scarce in recent years. We systematically evaluated the efficacy of probiotics on intermediate cardiovascular outcomes in patients with overweight or obesity. Methods: We searched for randomized controlled trials (RCTs) in four databases (until August 2021) that evaluated the effects of probiotics versus controls on intermediate cardiovascular outcomes. The outcomes were body mass index (BMI), weight, systolic blood pressure (SBP), diastolic blood pressure (DBP), glucose, low-density lipoprotein (LDL), and high-density lipoprotein (HDL) levels. Inverse variance random effects meta-analyses were used. The effects were reported as mean difference (MD), with their 95% confidence intervals (95% CI). The quality of evidence (QoE) was assessed with GRADE (grading of recommendations, assessment, development and evaluations) methodology. Results: A total of 25 RCTs were included (n = 2170), with a range of follow-up from two to six months. Probiotics likely reduced BMI (MD −0.27 kg/m^2^, 95%CI: −0.35 to −0.19; 17 RCTs; I2 = 26%, QoE: moderate), as well as likely reduced weight (MD −0.61 kg, 95%CI: −0.89 to −0.34; 15 RCTs; I2 = 0%, QoE: moderate), and may have slightly reduce LDL (MD −4.08 mg/dL; 95%CI: −6.99 to −1.17; 9 RCTs; I2 = 87%, QoE: low) in comparison to the controls. However, probiotics had no effect on SBP (MD −0.40 mmHg; 95%CI: −5.04 to 4.25; 7 RCTs; I2 = 100%, QoE: very low), DBP (MD −1.73 mmHg; 95%CI: −5.29 to 1.82; 5 RCTs; I2 = 98%, QoE: very low), glucose (MD −0.07 mg/dL; 95%CI −0.89 to 0.75; I2 = 96%, QoE: very low), HDL (MD −1.83 mg/dL; 95%CI: −4.14 to 2.47; 14 RCTs; I2 = 98%, QoE: very low), or triglycerides (MD −3.29 mg/dL, 95%CI −17.03 to 10.45; 14 RCTs, I2 = 95%, QoE: very low) compared to control arms, and the evidence was very uncertain. Conclusions: In obese or overweight patients, BMI, weight, and LDL were lower in patients who received probiotics compared to those who received controls. Other lipids, glucose, and blood pressure were not affected by the probiotics.

## 1. Introduction

Probiotics are microorganisms with beneficial potential for human health. Currently, there is literature supporting the idea that intestinal probiotics may exert effects outside the digestive system, including regulating energy balance, cardiovascular benefits, and mechanisms associated with the absorption and breakdown of intestinal contents [1,2,3,4]. In addition, there are some probiotic strains that decrease the translocation of microorganisms and improve intestinal barrier function by reducing the release of proinflammatory cytokines [5,6].

Obesity has been identified as a critical global problem [7]. In the physiological context, obesity is complex because there are several intrinsic and extrinsic factors to be considered, as well as genetics, diet, and other nutrigenomic factors. Some studies have mentioned that the gut microbiota has potential influence on the development of obesity. This is attributed to several mechanisms involving intestinal permeability and metabolic endotoxemia. In addition, a high-fat diet is closely associated with abdominal fat deposition and altered gut microbiota [8,9]. Furthermore, the intestinal microbiota is associated with the inflammatory process, insulin resistance, and type 2 diabetes mellitus. Intestinal microbiota is therefore considered a target in the treatment of diabetes and in the prevention of other cardiovascular diseases [9,10,11]. 

Recent literature has associated the development of obesity with an alteration in the intestinal microbiota (dysbiosis), which facilitates the storage of calories ingested in food. It is important to consider that there are certain intrinsic and extrinsic factors that can cause the imbalance of this intestinal ecosystem and which may lead not only to obesity, but also to the development of other alterations, such as insulin resistance. Some intervention studies show that oral administration of certain probiotics has a significant impact on some outcomes especially on body mass index (BMI) and weight control, suggesting a relationship between gut microbiota and body fat regulation [4,5,6,8]. For example, Firmicutes, Actinobacteria, Lactobacilli and Bifidobacterium are often related to these beneficial effects of probiotics [8,9,10,11].

We systematically evaluated the efficacy of probiotics on intermediate cardiovascular outcomes in patients with overweight or obesity.

## 2. Material and Methods

The PRISMA 2020 guidelines (Preferred Reporting Items for Systematic reviews and Meta-Analysis) were used for the writing and presentation of the present study [12]. In addition, this review was registered in PROSPERO (Prospective Registry of Systematic Reviews) (CRD42021264177).

### 2.1. Eligibility Criteria

We included studies that met the following inclusion criteria: (a) randomized controlled trials (RCTs) evaluating the effects of any dose and duration of probiotics on pre-defined intermediate cardiovascular outcomes; (b) a control group including milk, yogurt, maltodextrin, or placebo; and (c) evaluations adult patients (≥18 years) who were overweight (BMI 25 to 30 kg/m^2^) or obese (BMI > 30 kg/m^2^). Excluded studies were observational studies, case series, and case reports and commentaries, systematic reviews, conference abstracts, and editorials. The population included in this meta-analysis had no systemic history of hypertension or diabetes.

### 2.2. Search Methods

Electronic searches were conducted on 2 August 2021 in the Scopus, Web of Science, PubMed, and Embase search engines. We elaborated the search strategy using free text words and MeSH terms for PubMed, then adapted them according to the other databases. There were no language or publication date restrictions (Supplementary Table S1).

### 2.3. Outcomes 

Pre-defined intermediate cardiovascular outcomes were weight, BMI, systolic blood pressure (SBP), diastolic blood pressure (DBP), glucose, low-density lipoprotein (LDL), and high-density lipoprotein (HDL).

### 2.4. Selection and Data Collection of Studies

Study abstracts were downloaded to the Mendeley Reference Manager (Elsevier, Amsterdam, The Netherlands), and duplications were removed. The titles and abstracts were then independently reviewed by two authors (F.M.T. and C.D.A.). Subsequently, full-text articles were independently evaluated according to the selection criteria. All reasons for exclusion were recorded, and possible disagreements were resolved by consensus.

### 2.5. Data Extraction and Management

Data were extracted independently by two authors (F.M.T. and C.D.A.). An previously piloted extraction sheet was created in Microsoft Excel to record the author, year of publication, type of population (overweight, obese, both), mean age, proportion of diabetics and hypertensives, dose and duration of probiotic intervention, type of control, and outcomes for each intervention arm. Potential discrepancies were resolved by a third author (A.V.H.).

### 2.6. Assessment of Risk of Bias in Included Studies

To assess the risk of bias (RoB) of RCTs, the Cochrane RoB 2.0 tool was used [13]. Five domains of bias were assessed: randomization process, deviations from intended interventions, missing outcome data, outcome measurement, and selection of the reported outcome. Each bias domain was rated as “low,” “high,” or “some concerns.” Each RCT was then rated as being at low RoB, if all domains were at low RoB, high RoB, if at least one domain was at high RoB, or with some concerns of bias, if at least one domain was identified at some concerns of RoB, and no domain was at high RoB. Two review authors (F.M.T. and J.B.O.) independently conducted the assessments, and disagreements were resolved by consensus.

### 2.7. Data Synthesis Methods

Inverse variance random-effects meta-analyses were performed for all outcomes. The between-study variance was estimated using the Paule–Mandel method [14]. Effect measures were described as mean differences (MD) and their 95% confidence intervals (CI). The heterogeneity of effects among RCTs was described using the I^2^ statistic [15], with the following degrees: 0–30% (low), 30–60% (moderate), and >60% (high). Subgroup analyses by type of patient (overweight vs. obese vs. overweight/obese) and type of control (milk, yogurt, maltodextrin, or placebo) were conducted. The interaction test was considered statistically significant if the *p*-value was <0.10 [16]. The funnel plot and the Egger’s test were used to evaluate publication bias, only if ten or more RCTs were available. The metabin and metacont functions of the meta package of R 4.1.2 (www.r-project.org) (accessed on 7 March 2022) were used for all analyses. A two-tailed *p* < 0.05 was considered statistically significant.

For the evaluation of the quality of evidence (QoE), the GRADE methodology was used [17], evaluating five domains: inconsistency, risk of bias, imprecision, indirectness, and publication bias. Finally, QoE was presented in summary tables (SoF) using GRADEpro GDT (https://gradepro.org/, accessed on 7 July 2022, McMaster University and Evidence Prime, Inc. 2020) European Union Seventh Framework Programme (FP7—HEALTH.2010.3.1-1—two stage).

## 3. Results 

### 3.1. Selection of Studies

**Figure 1:**
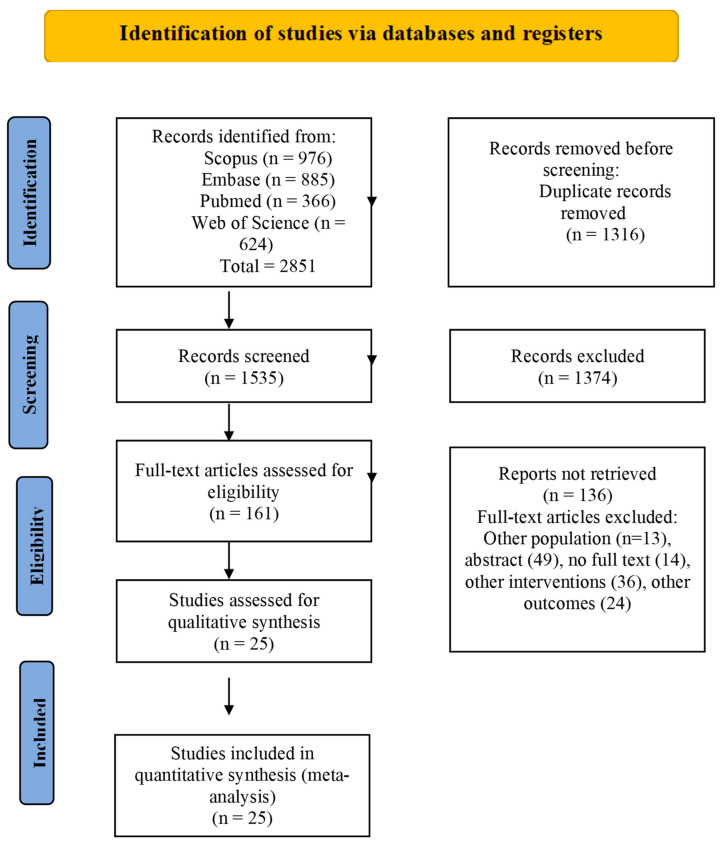
PRISMA Flow chart of the study selection process.

Of a total of 2851 abstracts, 1535 were available for evaluation, after removing duplicates. A total of 1374 records were excluded, and 161 full texts were further evaluated for inclusion. After excluding 136 studies after assessing populations, interventions, and outcomes that were out of the scope of our research question, we included 25 RCTs (n = 2170) in our study (Figure 1).

### 3.2. Characteristics of Included Trials

The studies included [18,19,20,21,22,23,24,25,26,27,28,29,30,31,32,33,34,35,36,37,38,39,40,41,42] in this systematic review were conducted in Denmark [18,20], Poland [19,30,40], USA [21], Iran [22,28,32,35,42], New Zealand [23,41], Korea [24,25,26,27], Malaysia [29], Japan [31], Indonesia [33], India [34,39], Canada [36], Russia [37], and Finland [38]. All RCTs had a follow-up period between 2 and 6 months. The study population was distributed across studies as follows: obesity [19,20,21,22,25,26,27,28,30,31,32,36,37,38,40,41,42], (n = 1603 patients), overweight [23,24,29,33,34,35,39], (n = 557 patients), and both overweight/obesity [18] (n = 70) (Table 1). The mean age range was between 28 and 68 years, there was no description of prevalence of diabetes, hypertension, or other cardiovascular diseases in individual RCTs. All included studies used probiotics of the bacterial genus (*Lactobacillus*, *Bifidobacterium*, *Streptococcus* and *Enterococcus*) [18,19,20,21,22,23,24,25,26,27,28,29,30,31,32,33,34,35,36,37,38,39,40,41,42]; control groups included placebo in 13 studies [19,21,22,24,25,26,27,29,30,34,35,36,37]; milk in four studies [18,23,31,33]; yogurt in two studies [28,42], and maltodextrin in six studies [20,32,38,39,40,41].

#### 3.2.1. Risk of Bias and Quality of Evidence

Only three RCTs were scored as high risks of bias [19,31,33]. Two RCTs had a high risk of bias in the measurement of the outcome [31,33]; one RCT had a high risk of bias in the selection of the reported result domain [19]. Moreover, 11 RCTs had some concerns of bias [19,22,23,28,29,30,35,36,37,38,41] (Supplementary Figure S1) [20,21,24,25,26,27,32,34,39,40,42]. The outcomes SBP, HDL, and triglycerides had very low QoE; DBP and LDL had low QoE; and BMI, weight, and glucose had moderate QoE (Table 2).

#### 3.2.2. Effect of Probiotics on Weight and Body Mass Index

**Figure 2:**
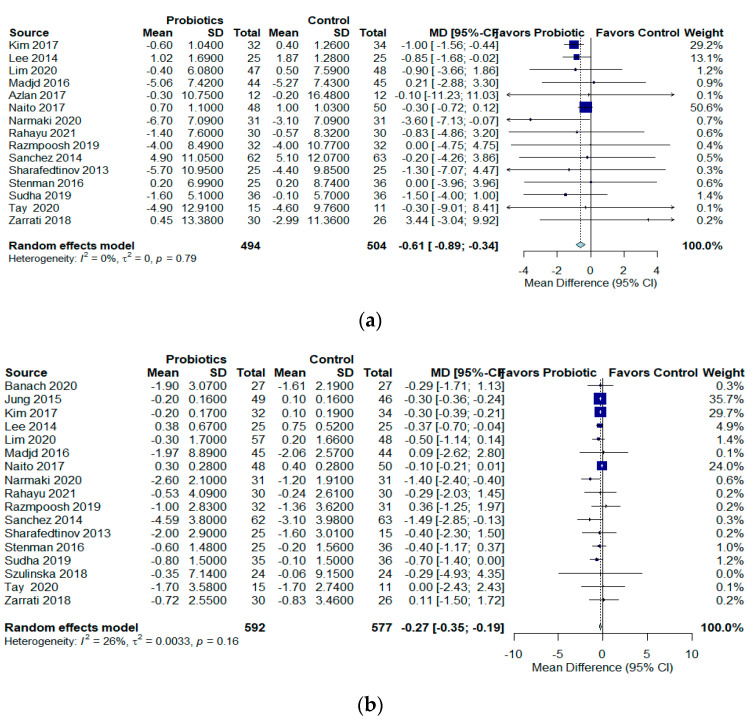
Effects of probiotics on (**a**) Weight in kg, and (**b**) BMI in kg/m^2^.

In 15 RCTs (n = 998) [25,26,27,28,29,31,32,33,35,36,37,38,39,41,42], probiotics likely reduces weight compared to the control group (MD −0.61 kg, 95% CI −0.89 to −0.34; I2 = 0%, QoE: moderate) (Figure 2a). In 17 RCTs (n = 1169) [19,24,25,26,28,31,32,33,35,36,37,38,39,40,41,42], probiotics likely reduced BMI compared to the control group (MD −0.27 kg/m^2^, 95% CI −0.35 to −0.19; I2 = 26%, QoE: moderate) (Figure 2b).

#### 3.2.3. Effect of Probiotics on Blood Pressure

**Figure 3:**
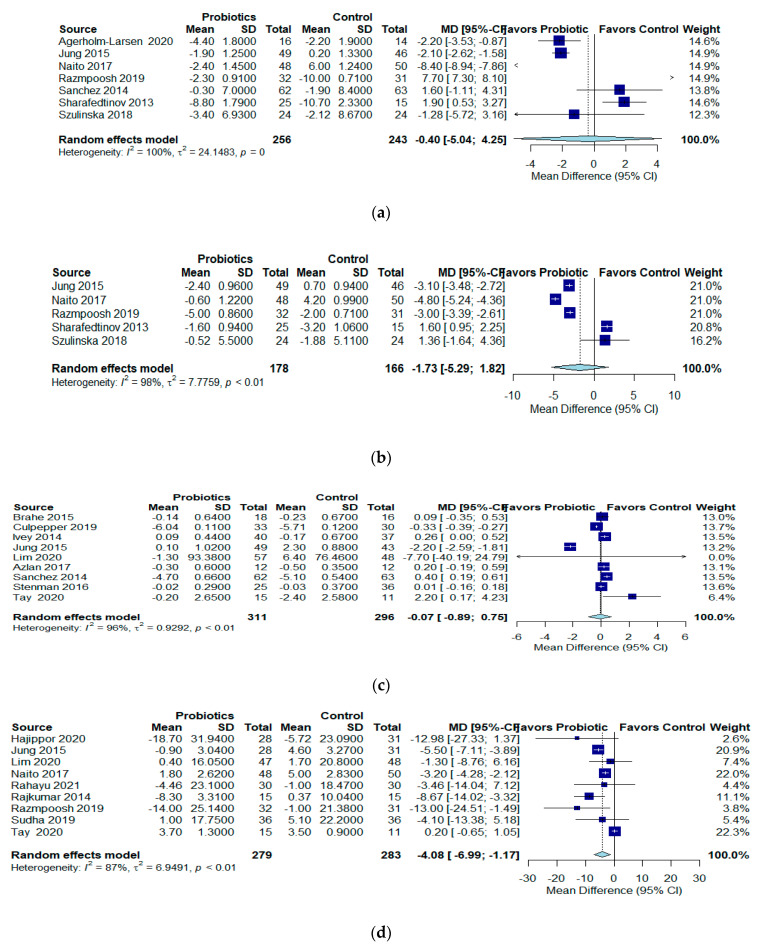

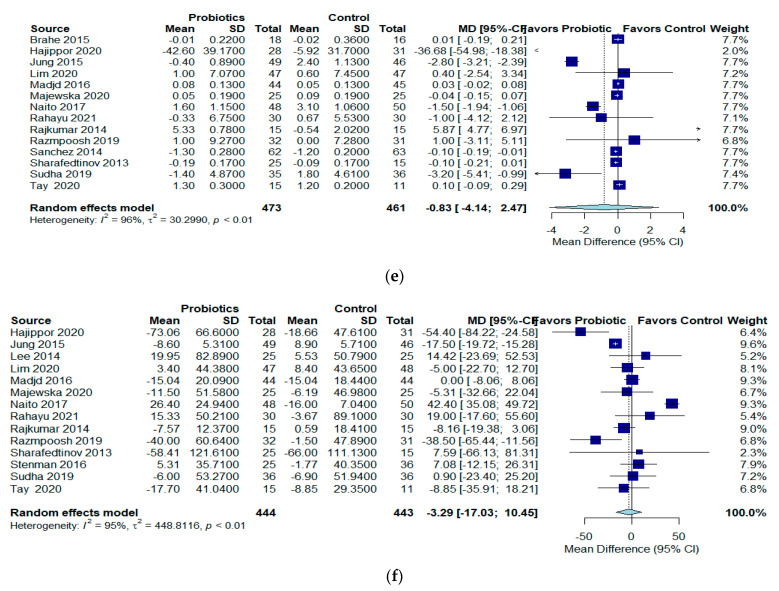
Effects of probiotics on: (**a**) SBP in mmHg; (**b**) DBP in mmHg; (**c**) glucose (mg/dL); (**d**) LDL (mg/dL); (**e**) HDL (mg/dL); and (**f**) triglycerides (mg/dL).

In seven RCTs (n = 499) [18,24,31,35,36,37,40], probiotics had no effect on SBP levels and controls (MD −0.40 mmHg; 95% CI −5.04 to 4.25; I2 = 100%, QoE: very low) (Figure 3a). In five RCTs (n = 344) [24,31,35,37,40], probiotics also had no effect on DBP levels and controls (MD −1.73 mmHg; 95% CI −5.29 to 1.82; I2 = 98%, QoE: very low) (Figure 3b). The evidence for SBP and DBP was very uncertain.

#### 3.2.4. Effect of Probiotics on Glucose

In nine RCTs (n = 607) [20,21,23,24,27,29,36,38,41] in overweight or obese patients, probiotics had no effect on mean glucose levels and controls (MD −0.07 mg/dL; 95%CI −0.89 to 0.75; I2 = 96%, QoE: very low) (Figure 3c), and the evidence was very uncertain.

#### 3.2.5. Effects of Probiotics on Lipids

In 9 RCTs (n = 562) [22,24,27,31,33,34,35,39,41] in overweight or obese patients, those who received probiotics reduce LDL slightly compared to controls (MD−4.08 mg/dL; 95% CI −6.99 to −1.17; I2 = 87%, QoE: low) (Figure 3d). In 14 RCTs (n = 934) [20,22,24,27,28,30,31,33,34,35,36,37,39,41] in overweight or obese patients, probiotics had no effect on HDL levels and controls (MD −0.83 mg/dL; 95% CI −4.14 to 2.47 mg/dL; I2 = 96%, QoE: very low) (Figure 3e). In 14 RCTs (n = 887) [22,24,26,27,28,30,31,33,34,35,37,38,39,41] in overweight or obese patients, probiotics had no effect on triglyceride levels (mg/dL) and controls (MD −3.29 mg/dL; 95% CI −17.03 to 10.45; I2 = 95%, QoE: very low) (Figure 3f). The evidence was very uncertain for lipids.

### 3.3. Subgroup Analyses

Subgroup analyses by type of control showed that probiotics significantly reduced BMI when the control group was placebo and maltodextrin (*p* for interaction <0.01); for DBP, when the control group was milk (*p* for interaction <0.01); for cholesterol and LDL, when the control group was placebo and milk (*p* for interaction <0.01 for both); and for HDL only when the control was milk (*p* for interaction <0.01) (Figures S2–S9). Subgroup analyses according to the type of patient showed that cholesterol and LDL were only reduced in overweight patients (*p* for interaction <0.01 and 0.03, respectively (Figures S10–S18). When analyzing the I^2^ by subgroups, it was found that the percentage of heterogeneity remained very high in most of the outcomes analyzed. However, only BMI and weight decreased when analyzed by type of control and type of patient.

## 4. Discussion

In our systematic review and meta-analysis, we found that overweight and/or obese patients receiving probiotics had lower weight, BMI, and LDL levels in comparison to those receiving controls. Other intermediate outcomes, such as SBP, DBP, glucose, HDL and triglycerides levels, were not significantly different between the probiotic and control arms. QoE for BMI, weight, and glucose was moderate, while other outcomes had low and very low QoE. Finally, our subgroup analysis by type of control showed that probiotics reduced BMI, when the control group was placebo and maltodextrin. For DBP, when the control group was milk; for cholesterol and LDL, when the control group was placebo and milk; and for HDL, only when the control was milk. On the other hand, our subgroup analyses according to patient type showed that cholesterol and LDL were only reduced in overweight patients.

Probiotics are defined as compounds containing certain microorganisms that will improve the “good” microbiota of the human body, especially when administered in adequate doses and frequencies. These probiotics can have beneficial effects on health when consumed on regular basis [43,44,45]. They are usually found naturally, although there are also some foods to which these probiotics are added to generate better accessibility for the population. Probiotics could help reducing unwanted immune responses, thus preventing chronic inflammation [29,46,47]. Among the main benefits of probiotics in obese people, studies described that they could reduce body weight during a follow-up period of 6 to 12 months [48]. In addition, some studies have shown that the consumption of probiotics reduced lipid levels. Some strains of probiotics have also been found to reduce insulin resistance [34,49,50].

A previous meta-analysis by Park et al. [51] in 2015 showed no effect of probiotic intake on body weight (MD −1.77 kg; 95% CI −4.84 to 1.29 kg) and BMI (MD 0.77 kg/m^2^; 95%CI −0.24 to 1.78 kg/m^2^). The authors included four placebo-controlled RCTs (n = 9) until 28 December 2014, searched in PubMed, Cochrane Library, and EMBASE search engines, and this study was limited to research in humans, without language restriction, and considered randomized clinical trial type studies with probiotic supplementation intervention without restriction in dose or route of administration, and as a control, placebo or no intervention was used. Additionally, this study used the old 2011 RoB tool for RCTs and did not assess the QoE per GRADE.

In contrast, in 2018, Borgeraas et al. [52], using 15 placebo-controlled RCTs (n = 15), found that probiotic intake had a small important effect on body weight (MD −0.60 kg; 95% CI −1.19 to −0.01 kg) and BMI (MD −0.27 kg/m^2^; 95%CI −0.45 to −0.08 kg/m^2^). The authors searched RCTs until September 1, 2016, using Medline and EMBASE engines, and they included randomized controlled trials in adult patients who were overweight (BMI 25–29.9 kg/m^2^) and obese (BMI ≥ 30 kg/m^2^). However, they excluded patients with gastrointestinal disorders, as well as studies involving pregnant women. Other limitations included the absence of QoE evaluation and the assessment of a small set of outcomes. The discrepancy in the times established for the evaluation of the effect of probiotics could be an important factor influencing the results reported by these authors. Finally, the 2016 study by Nikbakht et al. [53] in RCTs (n = 18) found that the reduction in blood glucose in the probiotic group was a trivial effect (MD −0.18 mmol/L; 95%CI −0.37 to 0.00 mmol/L). The authors searched information until February 2015 in PubMed (MEDLINE), Scopus, Cochrane Library, and the Cumulative Index to Nursing and Allied Health Literature (CINAHL) search engines, and they evaluated randomized or quasi-experimental (non-randomized controlled trials), full-text, English-language, controlled trials investigating the efficacy of probiotics or synbiotics in adults (age ≥ 18 years); they did not evaluate the certainty of the evidence.

Our meta-analysis had several strengths. First, we conducted a comprehensive search of four engines until August 2021, this being the most recent systematic review in contrast to those in previous studies. Second, we also used the most updated version of the RoB tool, the Cochrane RoB 2.0 tool, which was not used previously. Third, QoE per outcome was performed using GRADE methodology, which improved the understanding the strength of the probiotic effects. Fourth, we performed subgroup analyses in populations that may have differential effects of probiotics, in particular the type of patients and the types of controls. Finally, although we found statistically significant effects of probiotics on weight, BMI, and LDL levels, the absolute reductions are small and probably not clinically meaningful.

The present study had several limitations. First, a high heterogeneity of effects exists in regards to several outcomes, which may be due to methodological heterogeneity across the RCTs. We performed subgroup analyses by type of patient and type of control and found some effect of differences with respect to the main analyses, according to the type of controls. Second, most of the studies are from the Middle East and the East, so our findings may not be extrapolated to other populations, such as those in Latin America, North America, and Europe. Third, according to the GRADE methodology, QoE was very low for some intermediate outcomes due to the imprecision in some effects and the high risk of bias in some RCTs. Nonetheless, small important effects were found on weight and BMI, with moderate QoE. Fourth, clinical outcomes, such as mortality, myocardial infarction, and stroke, among others, were not evaluated in our systematic review, as these are scarce or not reported in the short period of follow-up of the included RCTs. Finally, the follow-up time across RCTs was short since most studies had an average follow-up of 6 months. Therefore, we could not evaluate the long-term effects of probiotics on our included studies.

## 5. Conclusions

In our systematic review of RCTs in overweight and obese populations, probiotics reduced BMI, weight, and LDL levels compared to placebo or other active controls, with a moderate to low quality of evidence. However, these effects were small in absolute terms and may not translate into clinically significant effects, indicating that the above findings should be taken with caution. Large RCTs with longer follow up are needed to evaluate the long-term effect of the intake of probiotics on intermediate cardiovascular outcomes and preferably, on clinical outcomes.

## Figures and Tables

**Table 1:** Characteristics of included studies.

Author, Year	Country	Sample Size	Population	Age	Intervention	Control	Outcomes	Follow-Up (Month)
Agerholm-Larsen et al., 2020 [18]	Denmark	70	Overweight 10% and Obese 90%	38.6 ± 2.1	*Enterococcus faecium* (human species) and two strains of *Streptococcus thermophilus*. The subjects attended the department 3 days a week (mornings or afternoons) to consume 300 mL yogurt or one placebo tablet and to collect products for consumption at home.	The placebo milk product was of identical composition as the other milk products, but chemically fermented with an organic acid (delta-acid-lactone) instead of a living bacterial culture.	SBP	2
Banach et al., 2020 [19]	Poland	54	Obese	34.8 ± 9.2	*Lactobacillus acidophilus* LA-5 and *Bifidobacterium lactis* BB-12 strains	Hypocaloric diet without deliberates	BMI	3
Brahe et al., 2015 [20]	Denmark	58	Obese	61.4 ± 6.5	*L. paracasei* F19	Maltodextrin	Glucose, HDL	1.5
Culpepper et al., 2019 [21]	USA	103	Obese	51.2 ± 1.4	*Bacillus subtilis* R0179, *Lactobacillus plantarum* HA-119, *Bifidobacterium animalis subsp. lactis* B94	Placebo (potato starch)	Glucose	4.5
Hajippor et al., 2020 [22]	Iran	140	Obese	40.9 ± 6.7	*Lactobacillus Acidophilus La-B5* and *Bifidobacterium lactis Bb-12* (at levels of colony-forming 4 × 107)	Vitamin D	Cholesterol, HDL, LDL, Triglycerides.	2.5
Ivey et al., 2014 [23]	New Zeland	156	Overweight	68.4 ± 7.8	*Lactobacillus acidophilus La5* and *Bifidobacterium animalis* subsp lactis Bb12	Control milk (prepared by Harvey Fresh, Harvey, WA, Australia)	Glucose	1.5
Jung et al., 2015 [24]	Korea	95	Overwight	40.1 ± 1.4	*L. curvatus* HY7601 and *L. plantarum* KY1032	The same amountof powder that did not contain any probiotics.	BMI, glucose, SBP, DBP, cholesterol, LDL, HDL and triglycerides	3
Kim et al., 2017 [25]	Korea	60	Obese	37.9	*Lactobacillus curvatus (L. curvatus*) HY7601 and *Lactobacillus plantarum (L. plantarum)* KY1032	Placebo	BMI, weight	3
Lee et al., 2014 [26]	Korea	50	Obese		*Streptococcus thermophiles* (KCTC 11870BP), *Lactobacillus plantarum* (KCTC 10782BP), *Lactobacillus acidophilus* (KCTC 11906BP), *Lactobacillus rhamnosus* (KCTC 12202BP), *Bifidobacterium lactis* (KCTC 11904BP), *Bifidobacterium longum* (KCTC 12200BP), and *Bifidobacterium breve* (KCTC 12201BP).	Placebo	BMI, weight, cholesterol, triglycerides	2
Lim et al., 2020 [27]	Korea	95	Obese	46.4 ± 12.2	*L. sakei* CJLS03	Placebo	BMI, weight, glucose, cholesterol, HDL, LDL, triglycerides	3
Madjd et al., 2016 [28]	Iran	89	Obese	32.2 ± 6.9	*Lactobacillus acidophilus* LA5) and *bifidobacteria* (*Bifidobacterium lactis* BB12)	Simple yogurt	BMI, weight, HDL, triglycerides	3
Azlan et al., 2017 [29]	Malaysia	24	Overweight	28.0 ± 8.3	*Lactobacillus acidophilus*, *Lactobacillus lactis*, *Lactobacillus casei*, *Bifi dobacterium longum, Bifi dobacterium bifi dum*, and *Bifi dobacterium* infantis	Hexbio^®^ B-Crobes Laboratory Sdn Bhd. Ipoh, Malaysia provided the MCP supplement and placebo samples.	Weight, glucose,	1
Majewska et al., 2020 [30]	Poland	50	Obese	55.2 ± 6.9	*Bifidobacterium bifidum* W23, *Bifidobacterium lactis* W51, *Bifidobacterium lactis* W52, *Lactobacillus acidophilus* W37, *Lactobacillus brevis* W63, *Lactobacillus casei* W56, *Lactobacillus salivarius* W24, *Lactococcus lactis* W19, and *Lactococcus lactis* W58	Placebo	HDL, triglycerides	3
Naito et al., 2017 [31]	Japan	248	Obese	46.6 ± 1.1	*Lactobacillus casei* strain *Shirota* (LcS)	Placebo milk	BMI, weight, SBP, DBP, cholesterol, LDL, HDL, triglycerides	3
Narmaki et al., 2020 [32]	Iran	62	Obese	35.2 ± 5.7	*Lactobacillus acidophilus* (1.8 × 10^9^ CFU/capsule), *Bifidobacterium bifidum* (1.8 × 10^9^ CFU/capsule), *Bifidobacterium lactis* (1.8 × 10^9^ CFU/capsule), *Bifidobacterium longum* (1.8 × 10^9^ FU/capsule), *Lactobacillus rhamnosus* (1 × 10^9^ CFU/capsule), *Lactobacillus reuteri* (1 × 10^9^ CFU/capsule)	Magnesium stearate, and maltodextrin	BMI, weight	3
Rahayu et al., 2021 [33]	Indonesia	60	Overweight	44.0 ± 6.2	*Lactobacillus plantarum* Dad-13	Skim milk obtained from a local supermarket was used in the placebo group.	BMI, weight, cholesterol, HDL, LDL, triglycerides	3
Rajkumar et al., 2014 [34]	India	60	Overweight	49(40–60)	Bifidobacteria (*Bifidobacterium longum*, *Bifidobacterium infantis*, and *Bifidobacterium breve*), four strains of lactobacilli (*Lactobacillus acidophilus*, *Lactobacillus paracasei, Lactobacillus delbrueckii* subsp. *bulgaricus*, and *Lactobacillus plantarum*), and one strain of *Streptococcus salivarius subsp. thermophilus*.	Omega 3	Cholesterol, HDL, LDL, triglycerides	1.5
Razmpoosh et al., 2019 [35]	Iran	70	Overweight	35.0 ± 10.0	*L. acidophilus* La5 and 1.79 106 CFU/g of B. lactis Bb12	Low energy diet	BMI, weight, SBP, DBP, cholesterol, HDL, LDL, triglycerides	2
Sanchez et al., 2014 [36]	Canada	153	Obese	37.0 ± 10.0	*Lactobacillus rhamnosus* CGMCC1.3724	Oligofructose and inulin	BMI, weight, glusoce, SBP, HDL	6
Sharafedtinov et al., 2013 [37]	Russia	40	Obese		*L. plantarum* TENSIA	Cheese	BMI, weight, SBP, DBP, HDL, triglycerides	1
Stenman et al., 2016 [38]	Finland	172	Obese	48.8 ± 10.5	*Bifidobacterium animalis* ssp. Lactis	Microcrystalline cellulose	BMI, weight, glucose, triglycerides	6
Sudha et al., 2019 [39]	India	92	Overweight	43.5	*Lactobacillus salivarius* UBLS-22, *Lactobacillus casei* UBLC-42, *Lactobacillus plantarum*, UBLP-40, *Lactobacillus acidophilus* UBLA-34, *Bifidobacterium breve* UBBr-01, and *Bacillus coagulans*	Maltodextrin	BMI, weight, cholesterol, LDL, HDL, triglycerides	3
Szulinska et al., 2018 [40]		110	Obese	55.1 ± 6.8	*Bifidobacterium bifidum* W23, *Bifidobacterium lactis* W51, *Bifidobacterium lactis* W52, *Lactobacillus acidophilus* W37, *Lactobacillus brevis* W63, *Lactobacillus casei* W56, *Lactobacillus salivarius* W24, *Lactococcus lactis* W19, and *Lactococcus lactis* W58	Maize starch and maltodextrins	BMI, SBP, DBP	3
Tay et al., 2020 [41]	New Zeland	59	Obese	52.9 ± 8.7	*Lacticaseibacillus rhamnosus*	Microcrystalline cellulose and dextrose anhydrate	BMI, weight, glucose, cholesterol, LDL, HDL, triglycerides	3
Zarrati et al., 2018 [42]	Iran	60	Obese	36 ± 8.4	*Lactobacillus acidophilus* La5, *Bifidobacterium* BB12, and *Lactobacillus casei*	Conventional yogurts	BMI, weight	2

**Table 2:** GRADE summary of findings table.

Outcomes	Anticipated Absolute Effects * (95% CI)		No of Participants (Studies)	Certainty of the Evidence (Grade)
	Risk with Control	Risk with Probiotics		
Body mass index follow-up: range 2 months to 6 months	The mean body mass index was 0.73 kg/m^2^.	MD 0.27 kg/m^2^ lower (0.35 lower to 0.19 lower)	1169 (17 RCTs)	⨁⨁⨁◯ Moderate ^a^
Weight follow-up: range 2 months to 6 months	The mean weight was −1.07 Kg.	MD 0.61 Kg lower (0.89 lower to 0.34 lower)	998 (15 RCTs)	⨁⨁⨁◯ Moderate ^b^
Systolic blood pressure follow-up: range 2 months to 6 months	The mean systolic blood pressure was −2.96 mmHg.	MD 0.4 mmHg lower (5.04 lower to 4.25 higher)	499 (7 RCTs)	⨁◯◯◯ Very low ^c,d,e^
Diastolic blood pressure follow-up: range 2 months to 6 months	The mean diastolic blood pressure was −0.43 mmHg.	MD 1.73 mmHg lower (5.29 lower to 1.82 higher)	344 (5 RCTs)	⨁◯◯◯ Very Low ^f,g,h^
Glucose follow-up: range 2 to 6 months	The mean glucose was −0.60 mg/dL.	MD 0.07 mg/dL lower (0.89 lower to 0.75 higher)	607 (9 RCTs)	⨁◯◯◯ Very Low ^i,j,k^
Low-density lipoprotein follow-up: range 2 months to 6 months	The mean low-density lipoprotein was 1.39 mg/dL.	MD 4.08 mg/dL lower (6.99 lower to 1.17 lower)	562 (9 RCTs)	⨁⨁◯◯ Low ^l,m^
High-density lipoprotein follow-up: range 2 months to 6 months	The mean high-density lipoprotein was 0.15 mg/dL.	MD 0.83 mg/dL lower (4.14 lower to 2.47 higher)	934 (14 RCTs)	⨁◯◯◯ Very low ^n,o,p^
Triglycerides follow-up: range 2 months to 6 months	The mean triglycerides was −8.65 mg/dL.	MD 3.29 mg/dL lower (17.03 lower to 10.45 higher)	887 (14 RCTs)	⨁◯◯◯ Very low ^q,r,s^
* The risk in the intervention group (and its 95% confidence interval) is based on the assumed risk in the comparison group and the relative effect of the intervention (and its 95% CI). The crosses are symbols marked according to GRADE methodology. CI: confidence interval; MD: mean difference				
GRADE working group grades of evidence High certainty: we are very confident that the true effect lies close to that of the estimate of the effect. Moderate certainty: we are moderately confident in the effect estimate: the true effect is likely to be close to the estimate of the effect, but there is a possibility that it is substantially different. Low certainty: our confidence in the effect estimate is limited: the true effect may be substantially different from the estimate of the effect. Very low certainty: we have very little confidence in the effect estimate: the true effect is likely to be substantially different from the estimate of the effect.				

Explanation: **a.** RoB 2.0: Banach et al. had a high risk of bias in the selection of the reported results, Madjd et al. had some concerns in the deviations from intended interventions and the selection of the reported result, Naito et al. had high risk of bias in the measurement of the outcome and some concerns in the selection of the reported result. Rahayu et al. had some concerns in the deviations from intended interventions and in the selection of the reported result, Razmpoosh et al., Sanchez et al., Sharafedtinov et al., Stenman et al., Szulinska et al., and Zarrati et al. had some concerns in the selection of the reported results. **b.** RoB 2.0: Agerholm-Larsen et al., Naito et al., Razmpoosh et al., Sanchez et al., Sharafedtinov et al. and Szulinska et al. had some concern about the risk of bias in some of the dimensions evaluated. **c.** RoB 2.0: Naito et al. had a high risk of bias in the measurement of the outcome and some concerns in the selection of the reported result, Razmpoosh et al., Sanchez et al., Sharafedtinov et al., and Szulinska et al. had some concerns in the selection of the reported results. **d.** Inconsistency: I^2^ = 100%. **e.** Imprecision: 95% CI of the effect was −5.04 to 4.25. **f.** RoB 2.0: Naito et al. had a high risk of bias in the measurement of the outcome and some concerns in the selection of the reported result. Razmpoosh et al., Sharafedtinov et al., and Szulinska et al. had some concerns in the selection of the reported results. **g.** Inconsistency: I^2^ = 98%. **h.** Imprecision: 95% CI of the effect was −5.29 to 1.82. **i.** RoB 2.0: Azlan et al. had some concerns in the randomization process, deviations from intended interventions, and the selection of the reported results. Sanchez et al. and Stenman et al. had some concerns in the selection of the reported results. **j.** Inconsistency: I^2^ = 96%. **k.** Imprecision: 95% CI of the effect was −0.89 to 0.75. **l.** RoB 2.0: Hajipoor et al. had some concern in the selection of the reported result, and Naito et al. had a high risk of bias in the measurement of the outcome and some concerns in the selection of the reported result. Rahayu et al. had some concerns in the deviations from intended interventions and in the selection of the reported result, and Razmpoosh et al., had some concerns in the selection of the reported results. **m.** Inconsistency: I^2^ = 87%. **n.** RoB 2.0: Hajippor et al. had some concerns in the selection of the reported result. Madjd et al. had some concerns in the deviations from intended interventions and the selection of the reported result. Majewska et al. had some concerns in the selection of the reported result. Naito et al. had a high risk of bias in the measurement of the outcome and some concerns in the selection of the reported result. Rahayu et al. had some concerns in the deviations from intended interventions and in the selection of the reported result, and Razmpoosh et al., Sanchez et al., and Sharafedtinov et al., had some concerns in the selection of the reported results. **o.** Inconsistency: I^2^ = 96%. **p.** Imprecision: 95% CI of the effect was −4.14 to 2.47. **q.** RoB 2.0: Hajippor et al. had some concerns in the selection of the reported result. Madjd et al. had some concerns in the deviations from intended interventions and the selection of the reported result. Majewska et al. had some concerns in the selection of the reported result. Naito et al. had a high risk of bias in the measurement of the outcome and some concerns in the selection of the reported result. Rahayu et al. had some concerns in the deviations from intended interventions and in the selection of the reported result, and Razmpoosh et al. and Sharafedtinov et al. had some concerns in the selection of the reported results. **r.** Inconsistency: I^2^ = 95%. **s.** Imprecision: 95% CI of the effect was −17.03 to 10.45.

## Data Availability

The data supporting this review were taken from previous studies. Data are available upon request to the corresponding author.

## Supplementary Materials

The following supporting information can be downloaded at: https://www.mdpi.com/article/10.3390/jcm12072554/s1.
