# Supplementary material for: Effects of Probiotics on Intermediate Cardiovascular Outcomes in Patients with Overweight or Obesity: A Systematic Review and Meta-Analysis
Source: J Clin Med. 2023 Mar 28;12(7):2554. doi: 10.3390/jcm12072554 (PMC10095238; doi:10.3390/jcm12072554)

Effects of probiotics on intermediate cardiovascular outcomes in patients with overweight or obesity: a systematic review and meta-analysis

Frank Mayta-Tovalino,[1] Carlos Diaz-Arocutipa,[1] Alejandro Piscoya,[1] Adrian V. Hernandez[1,4]

Supplementary contents

- **Supplemental Table S1:** Search strategies for PubMed, Scopus and Web of Science
- **Supplemental Figure S1:** Risk of bias assessment of included trials
- **Supplemental Figure S2:** Subgroup analyses by type of control on BMI
- **Supplemental Figure S3:** Subgroup analyses by type of control on glucose
- **Supplemental Figure S4:** Subgroup analyses by type of control on SBP
- **Supplemental Figure S5:** Subgroup analyses by type of control on DBP
- **Supplemental Figure S6:** Subgroup analyses by type of control on cholesterol
- **Supplemental Figure S7:** Subgroup analyses by type of control on LDL
- **Supplemental Figure S8:** Subgroup analyses by type of control on HDL
- **Supplemental Figure S9:** Subgroup analyses by type of control on triglycerides
- **Supplemental Figure S10:** Subgroup analyses by Overweight vs obese on BMI
- **Supplemental Figure S11:** Subgroup analyses by overweight vs obese on weight
- **Supplemental Figure S12:** Subgroup analyses by overweight vs obese on glucose
- **Supplemental Figure S13:** Subgroup analyses by overweight vs obese on SBP
- **Supplemental Figure S14:** Subgroup analyses by overweight vs obese on DBP
- **Supplemental Figure S15:** Subgroup analyses by overweight vs obese on cholesterol
- **Supplemental Figure S16:** Subgroup analyses by overweight vs obese on LDL
- **Supplemental Figure S17:** Subgroup analyses by overweight vs obese on HDL
- **Supplemental Figure S18:** Subgroup analyses by overweight vs obese on triglycerides.

Supplemental Table S1: Search strategies for PubMed, Scopus, and Web of Science

**PUBMED**
(prebiotics[mesh] OR probiotics[mesh] OR synbiotics[mesh] OR prebiotic*[tiab] OR probiotic*[tiab] OR synbiotic*[tiab] OR lactobacill*[tiab] OR lactococc*[tiab] OR lactobacillus[mesh] OR bifidobacter*[tiab] OR bifidobacterium[mesh] OR enterococc*[tiab] OR enterococcus[mesh] OR streptococc*[tiab] OR streptococcus[mesh] OR saccharomyces[tiab] OR saccharomyces[mesh] OR bacill*[tiab] OR bacillus[mesh] OR "escherichia coli"[tiab] OR "escherichia coli"[mesh]) AND (mortality[tiab] OR "myocardial infarction"[tiab] OR stroke[tiab] OR glucose[tiab] OR diabetes[tiab] OR weight[tiab] OR "body mass index"[tiab] OR BMI[tiab] OR hypertension[tiab] OR "blood pressure"[tiab] OR cholesterol[tiab] OR LDL[tiab] OR HDL[tiab] OR triglyceride*[tiab]) AND (overweight[mesh] OR overweight[tiab] OR obesity[mesh] OR obes*[tiab]) AND ("clinical trial"[tiab] OR random*[tiab] OR placebo[tiab])
**SCOPUS**
TITLE-ABS-KEY ((prebiotic* OR probiotic* OR synbiotic* OR lactobacill* OR lactococc* OR bifidobacter* OR enterococc* OR streptococc* OR saccharomyces OR bacill* OR "escherichia coli") AND (mortality OR "myocardial infarction" OR stroke OR glucose OR diabetes OR weight OR "body mass index" OR BMI OR hypertension OR "blood pressure" OR cholesterol OR LDL OR HDL OR triglyceride*) AND (overweight OR obes*) AND ("clinical trial" OR random* OR placebo))
**WOS**
TS=((prebiotic* OR probiotic* OR synbiotic* OR lactobacill* OR lactococc* OR bifidobacter* OR enterococc* OR streptococc* OR saccharomyces OR bacill* OR "escherichia coli") AND (mortality OR "myocardial infarction" OR stroke OR glucose OR diabetes OR weight OR "body mass index" OR BMI OR hypertension OR "blood pressure" OR cholesterol OR LDL OR HDL OR triglyceride*) AND (overweight OR obes*) AND ("clinical trial" OR random* OR placebo))
**EMBASE**
(prebiotic* OR probiotic* OR synbiotic* OR lactobacill*:ti,ab OR lactococc*:ti,ab OR bifidobacter*:ti,ab OR enterococc*:ti,ab OR streptococc*:ti,ab OR saccharomyces:ti,ab OR bacill*:ti,ab OR 'escherichia coli':ti,ab) AND (mortality:ti,ab OR 'myocardial infarction':ti,ab OR stroke:ti,ab OR glucose:ti,ab OR diabetes:ti,ab OR weight:ti,ab OR 'body mass index':ti,ab OR bmi:ti,ab OR hypertension:ti,ab OR 'blood pressure':ti,ab OR cholesterol:ti,ab OR ldl:ti,ab OR hdl:ti,ab OR triglyceride*:ti,ab) AND (overweight OR obes*) AND ('clinical trial' OR random* OR placebo)

Supplemental Figure S1: Risk of bias assessment of included trials

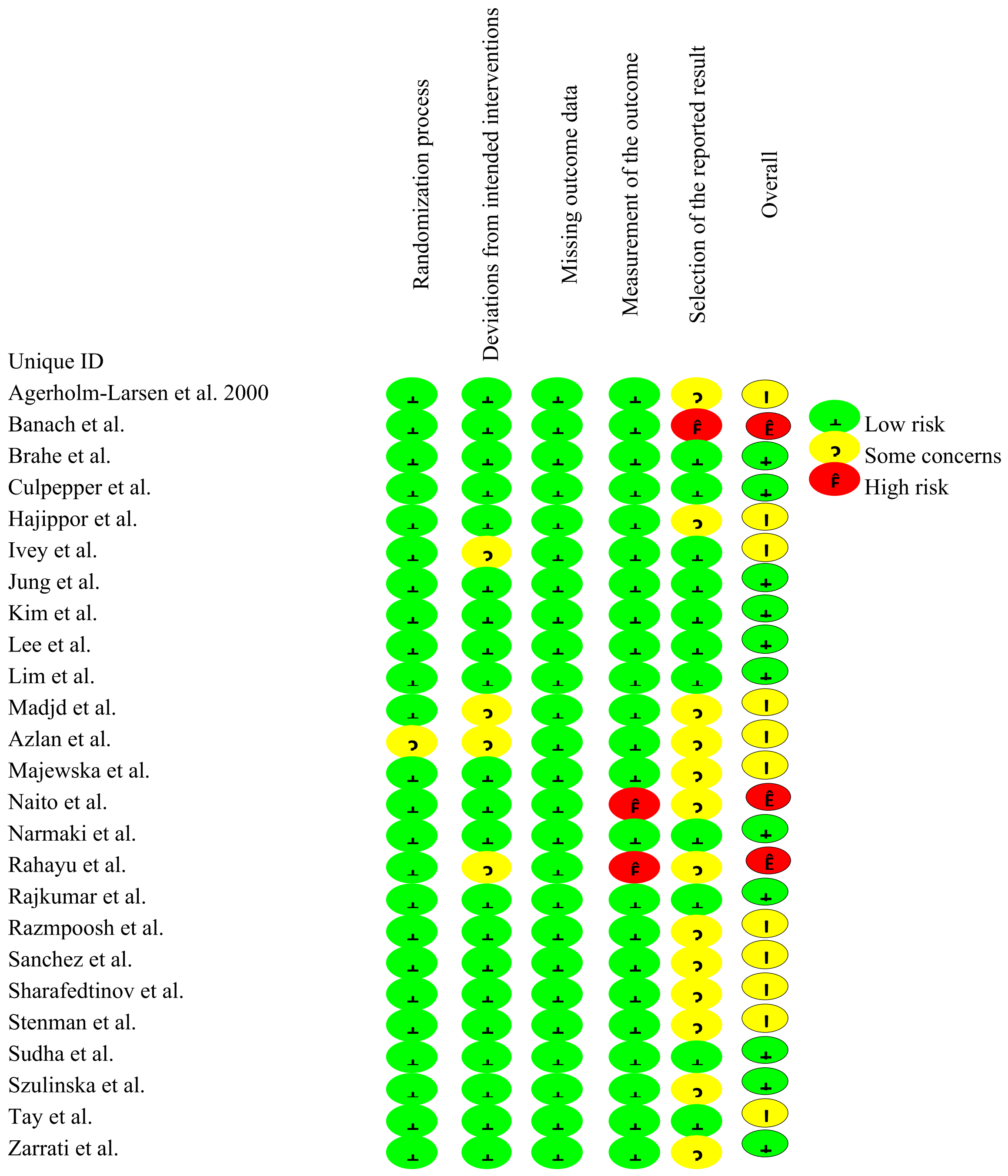

Supplemental Figure S2: Subgroup analyses by type of control on BMI

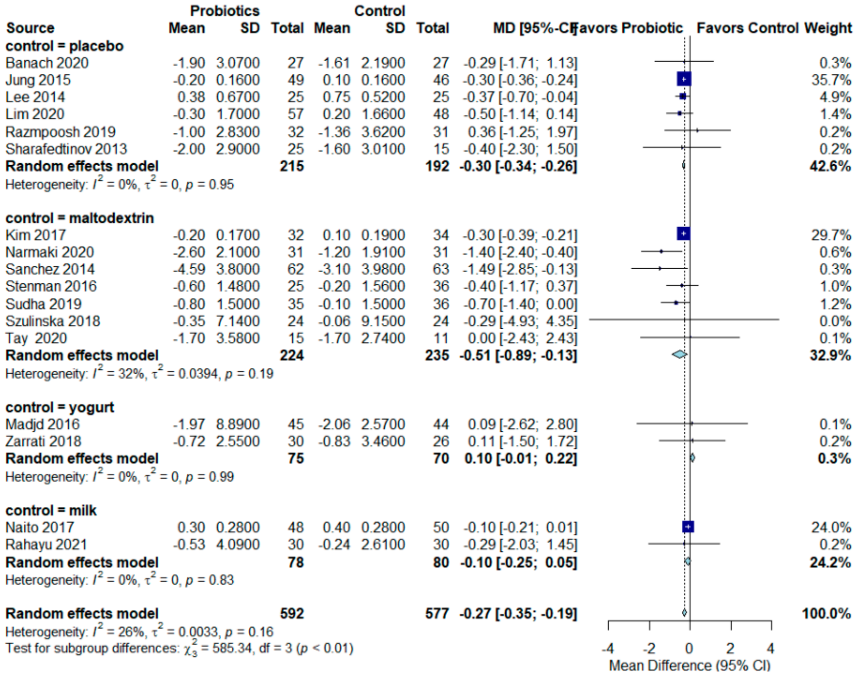

Supplemental Figure S3: Subgroup analyses by type of control on glucose

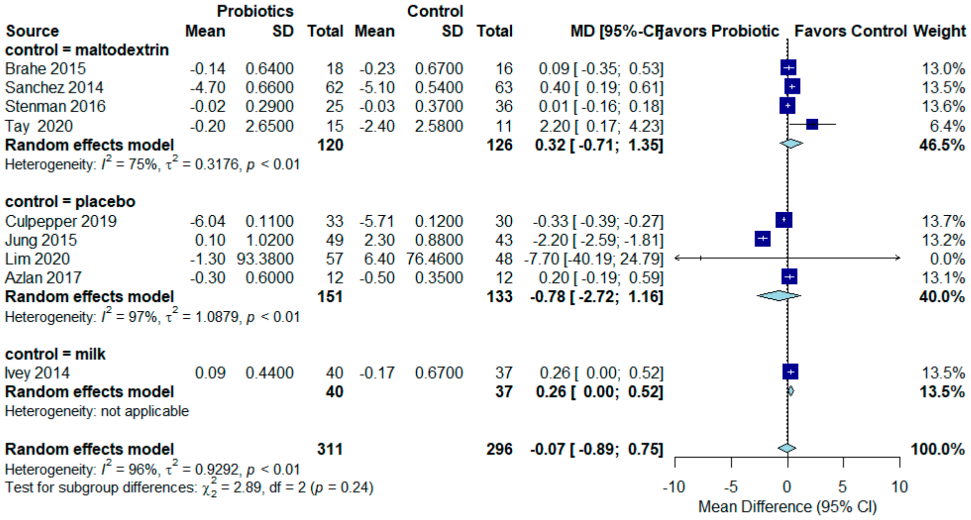

Supplemental Figure S4: Subgroup analyses by type of control on SBP

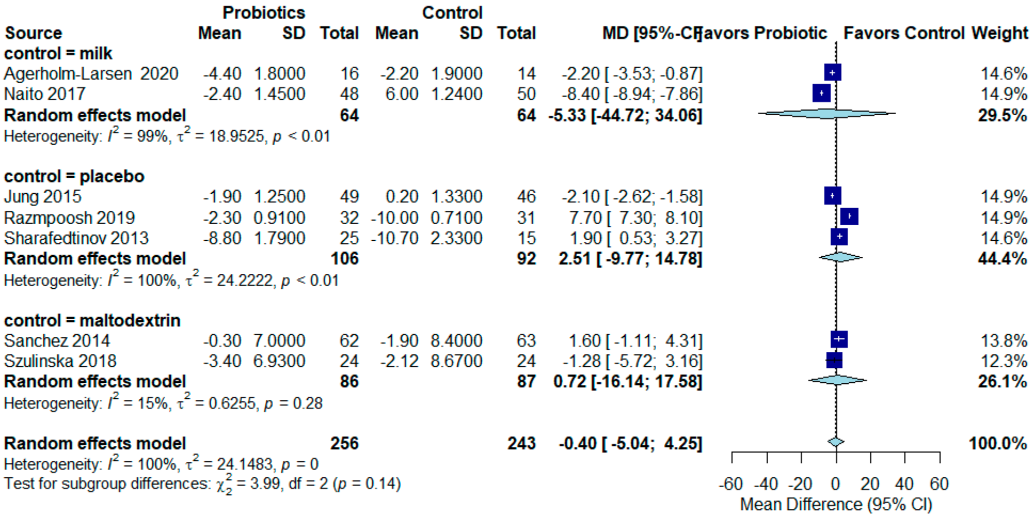

Supplemental Figure S5: Subgroup analyses by type of control on DBP

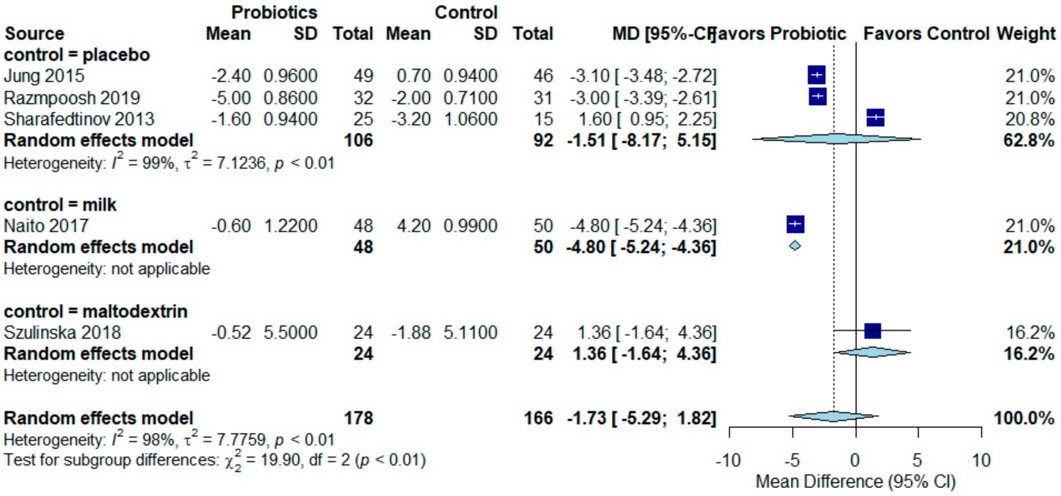

Supplemental Figure S6: Subgroup analyses by type of control on cholesterol

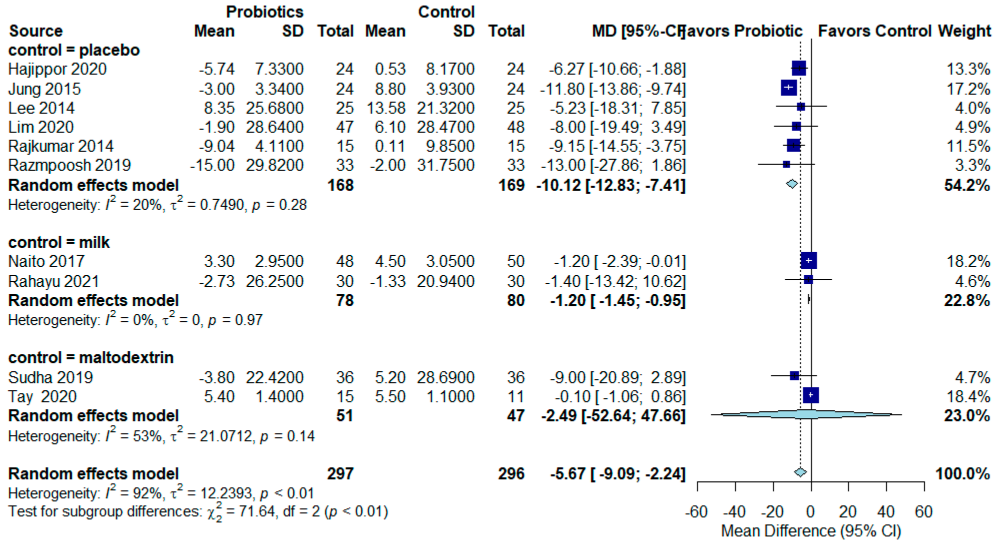

Supplemental Figure S7: Subgroup analyses by type of control on LDL

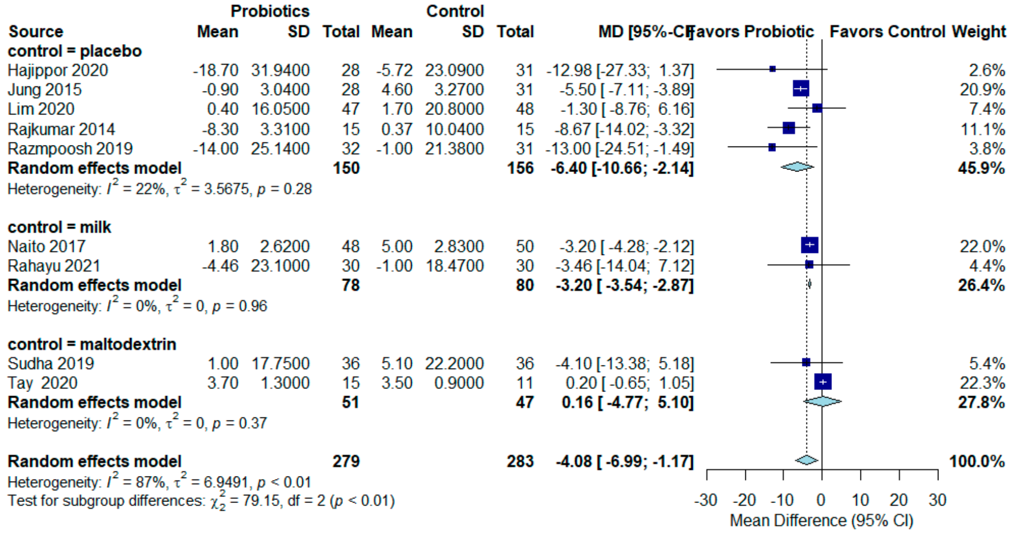

Supplemental Figure S8: Subgroup analyses by type of control on HDL

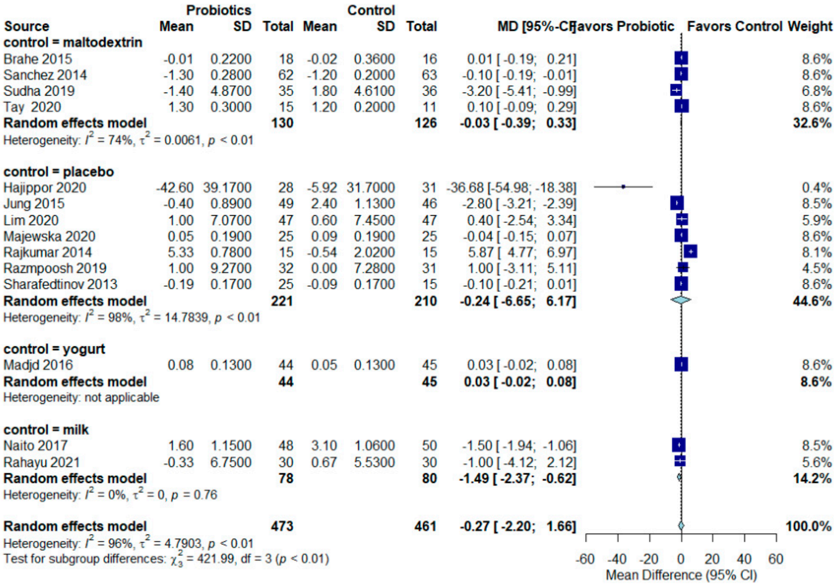

Supplemental Figure S9: Subgroup analyses by type of control on tryglicerides

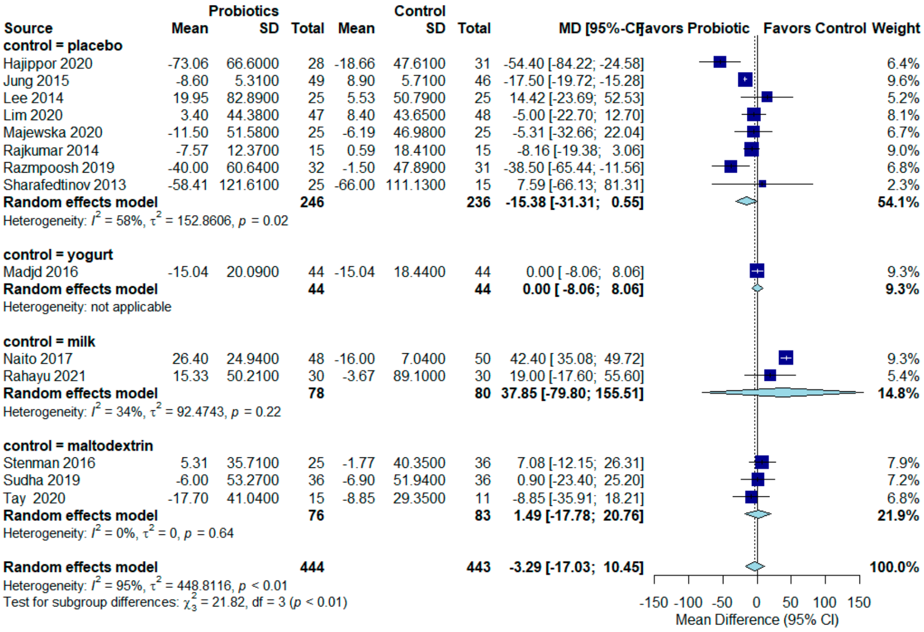

Supplemental Figure S10: Subgroup analyses by overweight vs obese on BMI

Source	Probiotics Mean	Probiotics SD	Probiotics Total	Control Mean	Control SD	Control Total	MD [95%-CI]	Weight
**obesity = obese**								
Banach 2020	-1.90	3.0700	27	-1.61	2.1900	27	-0.29 [-1.71; 1.13]	0.3%
Kim 2017	-0.20	0.1700	32	0.10	0.1900	34	-0.30 [-0.39; -0.21]	29.7%
Lee 2014	0.38	0.6700	25	0.75	0.5200	25	-0.37 [-0.70; -0.04]	4.9%
Lim 2020	-0.30	1.7000	57	0.20	1.6600	48	-0.50 [-1.14; 0.14]	1.4%
Madjd 2016	-1.97	8.8900	45	-2.06	2.5700	44	0.09 [-2.62; 2.80]	0.1%
Naito 2017	0.30	0.2800	48	0.40	0.2800	50	-0.10 [-0.21; 0.01]	24.0%
Narmaki 2020	-2.60	2.1000	31	-1.20	1.9100	31	-1.40 [-2.40; -0.40]	0.6%
Sanchez 2014	-4.59	3.8000	62	-3.10	3.9800	63	-1.49 [-2.85; -0.13]	0.3%
Sharafedtinov 2013	-2.00	2.9000	25	-1.60	3.0100	15	-0.40 [-2.30; 1.50]	0.2%
Stenman 2016	-0.60	1.4800	25	-0.20	1.5600	36	-0.40 [-1.17; 0.37]	1.0%
Szulinska 2018	-0.35	7.1400	24	-0.06	9.1500	24	-0.29 [-4.93; 4.35]	0.0%
Tay 2020	-1.70	3.5800	15	-1.70	2.7400	11	0.00 [-2.43; 2.43]	0.1%
Zarrati 2018	-0.72	2.5500	30	-0.83	3.4600	26	0.11 [-1.50; 1.72]	0.2%
**Random effects model**			**446**			**434**	**-0.26 [-0.40; -0.13]**	**62.7%**
Heterogeneity: $I^2 = 33\%$, $\tau^2 = 0.0068$, $p = 0.11$								
**obesity = overweight**								
Jung 2015	-0.20	0.1600	49	0.10	0.1600	46	-0.30 [-0.36; -0.24]	35.7%
Rahayu 2021	-0.53	4.0900	30	-0.24	2.6100	30	-0.29 [-2.03; 1.45]	0.2%
Razmpoosh 2019	-1.00	2.8300	32	-1.36	3.6200	31	0.36 [-1.25; 1.97]	0.2%
Sudha 2019	-0.80	1.5000	35	-0.10	1.5000	36	-0.70 [-1.40; 0.00]	1.2%
**Random effects model**			**146**			**143**	**-0.30 [-0.39; -0.22]**	**37.3%**
Heterogeneity: $I^2 = 0\%$, $\tau^2 = 0$, $p = 0.59$								
**Random effects model**			**592**			**577**	**-0.27 [-0.35; -0.19]**	**100.0%**
Heterogeneity: $I^2 = 26\%$, $\tau^2 = 0.0033$, $p = 0.16$								
Test for subgroup differences: $\chi^2_1 = 0.36$, df = 1 ($p = 0.55$)								

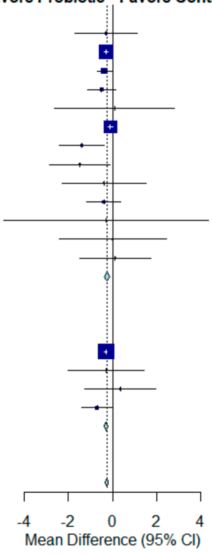

Supplemental Figure S11: Subgroup analyses by overweight vs obese on weight

Source	Probiotics Mean	Probiotics SD	Probiotics Total	Control Mean	Control SD	Control Total	MD [95%-CI]	Weight
**obesity = obese**								
Kim 2017	-0.60	1.0400	32	0.40	1.2600	34	-1.00 [-1.56; -0.44]	29.2%
Lee 2014	1.02	1.6900	25	1.87	1.2800	25	-0.85 [-1.68; -0.02]	13.1%
Lim 2020	-0.40	6.0800	47	0.50	7.5900	48	-0.90 [-3.66; 1.86]	1.2%
Madjd 2016	-5.06	7.4200	44	-5.27	7.4300	45	0.21 [-2.88; 3.30]	0.9%
Naito 2017	0.70	1.1000	48	1.00	1.0300	50	-0.30 [-0.72; 0.12]	50.6%
Narmaki 2020	-6.70	7.0900	31	-3.10	7.0900	31	-3.60 [-7.13; -0.07]	0.7%
Sanchez 2014	4.90	11.0500	62	5.10	12.0700	63	-0.20 [-4.26; 3.86]	0.5%
Sharafedtinov 2013	-5.70	10.9500	25	-4.40	9.8500	25	-1.30 [-7.07; 4.47]	0.3%
Stenman 2016	0.20	6.9900	25	0.20	8.7400	36	0.00 [-3.96; 3.96]	0.6%
Tay 2020	-4.90	12.9100	15	-4.60	9.7600	11	-0.30 [-9.01; 8.41]	0.1%
Zarrati 2018	0.45	13.3800	30	-2.99	11.3600	26	3.44 [-3.04; 9.92]	0.2%
**Random effects model**			**384**			**394**	**-0.60 [-0.93; -0.27]**	**97.5%**
Heterogeneity: $I^2 = 0\%$, $\tau^2 = 0$, $p = 0.53$								
**obesity = overweight**								
Azlan 2017	-0.30	10.7500	12	-0.20	16.4800	12	-0.10 [-11.23; 11.03]	0.1%
Rahayu 2021	-1.40	7.6000	30	-0.57	8.3200	30	-0.83 [-4.86; 3.20]	0.6%
Razmpoosh 2019	-4.00	8.4900	32	-4.00	10.7700	32	0.00 [-4.75; 4.75]	0.4%
Sudha 2019	-1.60	5.1000	36	-0.10	5.7000	36	-1.50 [-4.00; 1.00]	1.4%
**Random effects model**			**110**			**110**	**-1.07 [-2.13; 0.00]**	**2.5%**
Heterogeneity: $I^2 = 0\%$, $\tau^2 = 0$, $p = 0.95$								
**Random effects model**			**494**			**504**	**-0.61 [-0.89; -0.34]**	**100.0%**
Heterogeneity: $I^2 = 0\%$, $\tau^2 = 0$, $p = 0.79$								
Test for subgroup differences: $\chi^2_1 = 1.62$, df = 1 ($p = 0.20$)								

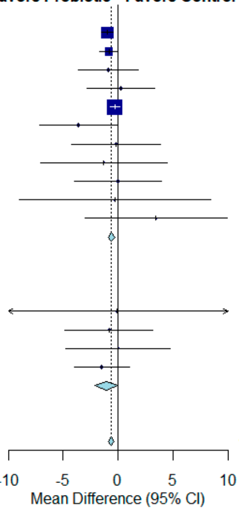

Supplemental Figure S12: Subgroup analyses by overweight vs obese on glucose

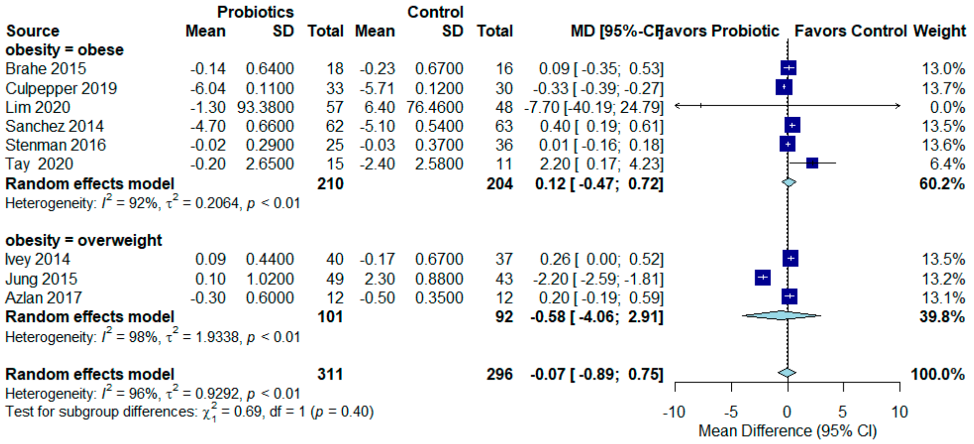

Supplemental Figure S13: Subgroup analyses by overweight vs obese on SBP

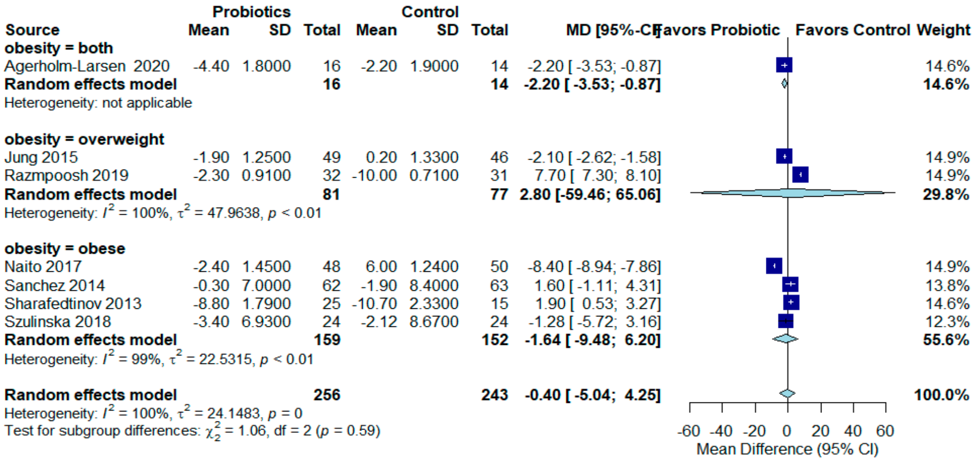

Supplemental Figure S14: Subgroup analyses by overweight vs obese on DBP

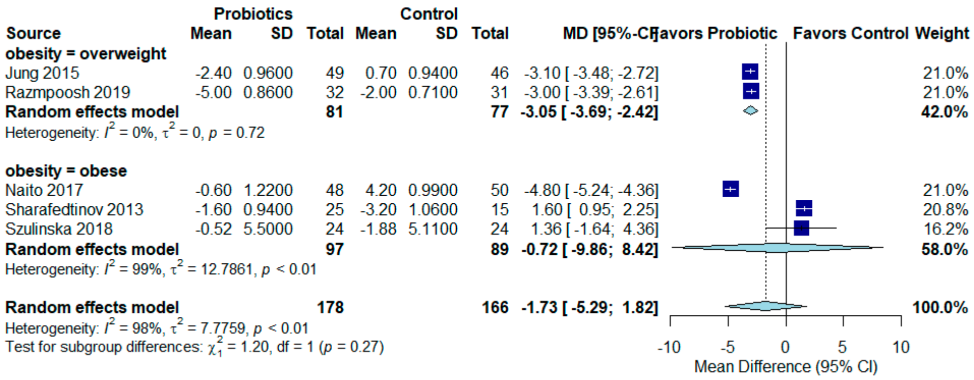

Supplemental Figure S15: Subgroup analyses by overweight vs obese on cholesterol

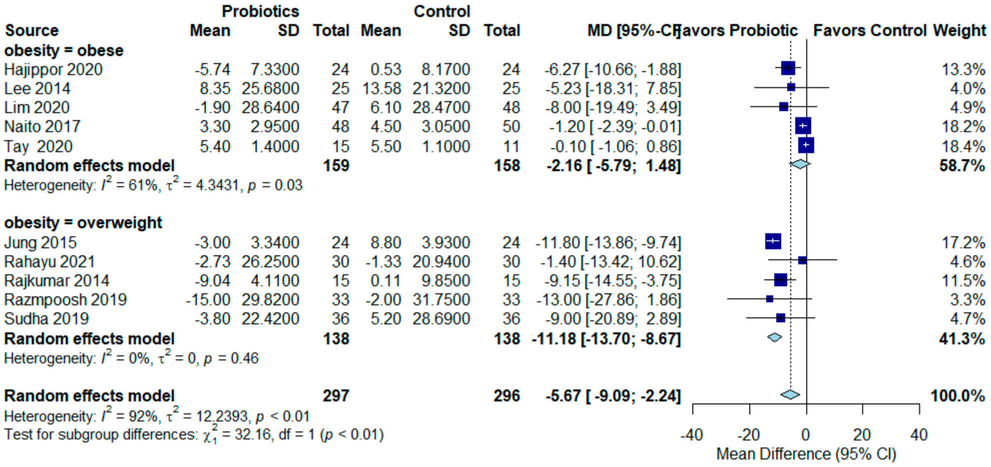

Supplemental Figure S16: Subgroup analyses by overweight vs obese on LDL

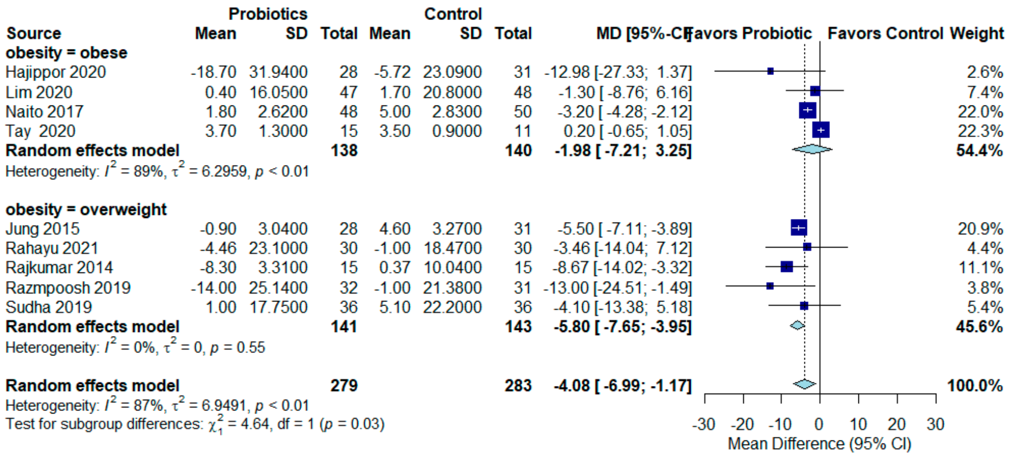

Supplemental Figure S17: Subgroup analyses by overweight vs obese on HDL

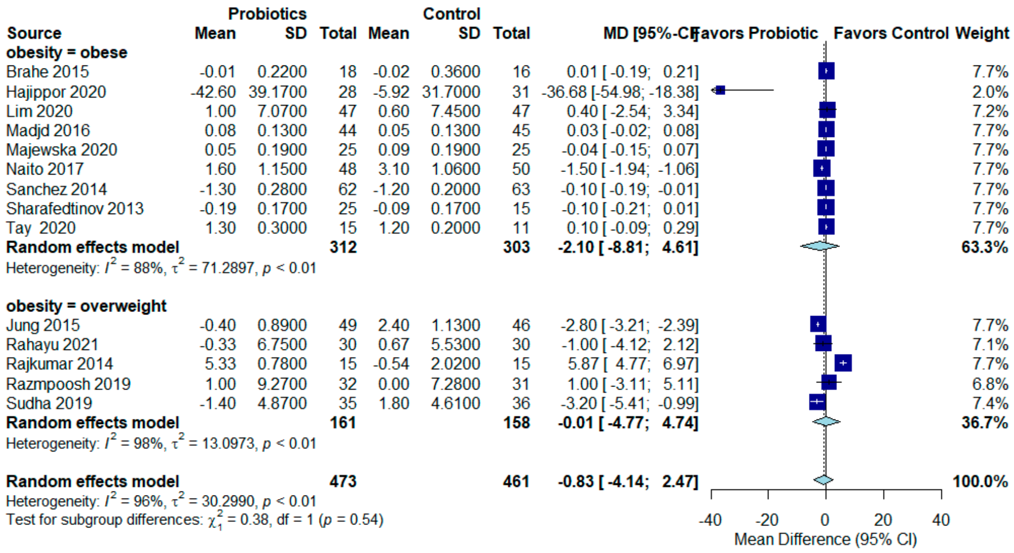

Supplemental Figure S18: Subgroup analyses by overweight vs obese on triglycerides

Source	Probiotics Mean	Probiotics SD	Probiotics Total	Control Mean	Control SD	Control Total	MD [95%-CI]	Weight
**obesity = obese**								
Hajippor 2020	-73.06	66.6000	28	-18.66	47.6100	31	-54.40 [-84.22; -24.58]	6.4%
Lee 2014	19.95	82.8900	25	5.53	50.7900	25	14.42 [-23.69; 52.53]	5.2%
Lim 2020	3.40	44.3800	47	8.40	43.6500	48	-5.00 [-22.70; 12.70]	8.1%
Madjd 2016	-15.04	20.0900	44	-15.04	18.4400	44	0.00 [-8.06; 8.06]	9.3%
Majewska 2020	-11.50	51.5800	25	-6.19	46.9800	25	-5.31 [-32.66; 22.04]	6.7%
Naito 2017	26.40	24.9400	48	-16.00	7.0400	50	42.40 [35.08; 49.72]	9.3%
Sharafedtinov 2013	-58.41	121.6100	25	-66.00	111.1300	15	7.59 [-66.13; 81.31]	2.3%
Stenman 2016	5.31	35.7100	25	-1.77	40.3500	36	7.08 [-12.15; 26.31]	7.9%
Tay 2020	-17.70	41.0400	15	-8.85	29.3500	11	-8.85 [-35.91; 18.21]	6.8%
**Random effects model**			**282**			**285**	**0.69 [-19.47; 20.85]**	**62.1%**
Heterogeneity: $I^2 = 92\%$, $\tau^2 = 568.1527$, $p < 0.01$								
**obesity = overweight**								
Jung 2015	-8.60	5.3100	49	8.90	5.7100	46	-17.50 [-19.72; -15.28]	9.6%
Rahayu 2021	15.33	50.2100	30	-3.67	89.1000	30	19.00 [-17.60; 55.60]	5.4%
Rajkumar 2014	-7.57	12.3700	15	0.59	18.4100	15	-8.16 [-19.38; 3.06]	9.0%
Razmpoosh 2019	-40.00	60.6400	32	-1.50	47.8900	31	-38.50 [-65.44; -11.56]	6.8%
Sudha 2019	-6.00	53.2700	36	-6.90	51.9400	36	0.90 [-23.40; 25.20]	7.2%
**Random effects model**			**162**			**158**	**-12.02 [-31.68; 7.64]**	**37.9%**
Heterogeneity: $I^2 = 63\%$, $\tau^2 = 89.3641$, $p = 0.03$								
**Random effects model**			**444**			**443**	**-3.29 [-17.03; 10.45]**	**100.0%**
Heterogeneity: $I^2 = 95\%$, $\tau^2 = 448.8116$, $p < 0.01$								
Test for subgroup differences: $\chi^2_1 = 1.28$, df = 1 ($p = 0.26$)								

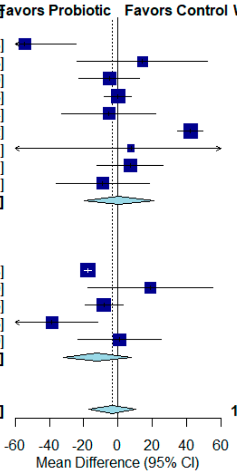

Supplement: Supplementary file 1 [file jcm-12-02554-s001.zip › jcm-2190585-supplementary.pdf]
